# Towards greater understanding of implementation during systematic reviews of complex healthcare interventions: the framework for implementation transferability applicability reporting (FITAR)

**DOI:** 10.1186/s12874-019-0723-y

**Published:** 2019-04-18

**Authors:** Susan Baxter, Maxine Johnson, Duncan Chambers, Anthea Sutton, Elizabeth Goyder, Andrew Booth

**Affiliations:** School of Health and Related Research, University of Sheffield, Regent Court, Regent Street, Sheffield, S14DA UK

**Keywords:** Applicability, Transferability, Systematic reviews, Checklist, Methodology

## Abstract

**Background:**

There have been calls for greater consideration of applicability and transferability in systematic reviews, to improve their usefulness in informing policy and practice. Understanding how evidence is, or is not applicable and transferable to varying local situations and contexts, is a key challenge for systematic review synthesis in healthcare. Assessing applicability and transferability in systematic reviews is reported to be difficult, particularly in reviews of complex interventions. There is a need for exploration of factors perceived to be important by policy-makers, and for further guidance on which items should be reported. In this paper we focus on the process of development of a framework that can be used by systematic reviewers to identify and report data across studies relating to applicability and transferability.

**Methods:**

The framework was developed by scrutinising existing literature on applicability and transferability, examining data during a systematic review of highly complex changes to health service delivery, and was informed by stakeholder engagement. The items of the framework were thus grounded in both data identified during a real review, and stakeholder input. The paper describes examples of data identified using the framework during a review of integrated care interventions, and outlines how it informed analysis and reporting of the review findings.

**Results:**

The Framework for Implementation Transferability Applicability Reporting (FITAR) comprises 44 items which can be used to structure analysis and reporting across studies during systematic reviews of complex interventions. The framework prompts detailed consideration of contextual data during extraction and reporting, within areas of: patient type and populations; type of organisations and systems; financial and commissioning processes; systems leadership elements; features of services; features of the workforce; and finally elements of the interventions/initiatives.

**Conclusions:**

Use of the framework during our review of complex healthcare interventions helped the review team to surface contextual data, which may not be commonly extracted, analysed and reported. Further exploration and evaluation of systems for identifying and reporting these factors during reviews is required.

**Electronic supplementary material:**

The online version of this article (10.1186/s12874-019-0723-y) contains supplementary material, which is available to authorized users.

## Background

Systematic reviews are a cornerstone of evidence-based healthcare to inform decisions about policy and practice. However, user concerns about the applicability of research findings to their own populations and setting represents a commonly reported barrier to their use [1]. Commentators have also articulated uncertainty regarding methods for implementing potentially effective interventions in local contexts [2]. Efforts to translate research findings into practice may therefore fail, if contextual factors which could affect implementation and outcomes are not analysed and reported [3].

Researchers recognise that the transfer of study findings to policy and practice is complex and multi-faceted. The PARiHS (Promoting Action on Research Implementation in Health Services) framework for example [4] highlights that some contexts are more conducive to successful implementation of evidence into practice than others. Authors have argued for a greater emphasis on generalisation and applicability in reporting [5]. This emphasis is needed to provide “real world” information about healthcare interventions [6].

There is a recognised lack of standardisation in usage of the terms applicability, generalisability, and transferability across the research literature. The terms “generalisability” and “applicability” are often considered to be synonymous. However, “generalisability” (synonymous with external validity) is usually used to refer to whether the *results* of a study might be relevant to other general sites and populations. Whereas “applicability” typically refers to feasibility and process, providing insights into whether and how an intervention may be *implemented* elsewhere in a particular *context* [2, 7]. The term “transferability” is similar to “generalisability” in referring to the likelihood of replication of *outcomes*, but in common with applicability, it is distinguished from generalisability by relating to outcomes in a specific *context* [1, 8]. Given their shared focus on local context, it has been recommended that applicability and transferability should be considered together [1].

Greater consideration of applicability and transferability in systematic reviews has been called for, [9] although review authors have noted insufficient reporting of relevant information in primary studies for their assessment [10]. While current guidance highlights the need to consider these aspects, further detail regarding appropriate methods to use would be beneficial. The PRISMA (Preferred Reporting Items for Systematic Reviews and Meta-Analyses) statement for example includes only one item relating to applicability; that the main findings should be considered in terms of “relevance to key groups” [11]. The GRADE rating system highlights that the review team should consider the “translation of the evidence into practice in a specific setting” [12]. Much of the focus of work by the Cochrane Collaboration, has been on the evaluation of internal validity, [13] and the importance of separating assessment of internal validity from that of external validity [14].

The current limited specification of methods may be associated with the reported difficulty in assessing applicability and transferability; in particular making decisions regarding which items are relevant and should be reported [10]. Reviews of complex interventions present particular challenges for considering applicability, due to: heterogeneity of target populations; multiple component interventions; varying duration and delivery; and outcome diversity [5].

In this paper we describe the methodological process which underpinned development of a tool to support the identification and reporting of data relating to applicability and transferability across studies. The creation of the framework was data-driven, and informed by stakeholder input at all stages. The paper describes the development, and experiences of using the tool, drawing on examples from a systematic review of complex healthcare interventions. We intended that the framework we developed would complement other established methods used during the systematic review process. We envisaged that the framework would assist with identification of relevant data during data extraction, and aid analysis and interpretation across studies during synthesis and reporting of the review findings.

## Methods

The method for development of our framework was informed by three complementary activities: review of existing methodological literature; analysis and re-analysis of data extracted during an exemplar review; and stakeholder engagement. All three elements contributed to development via an iterative process.

We initially carried out a review of the literature to search for relevant studies and existing tools. We made the decision not to carry out a systematic review, as we identified other existing relevant reviews [1, 15]. We drew on these authors’ search strategy to carry out a supplementary search of MEDLINE and Google Scholar in order to check for any additional relevant papers reporting methods for evaluation or reporting of applicability during primary or secondary research studies. We examined studies which had been included in the previous reviews.

Many existing tools identified were based on the established PICO/PICOC (population, intervention, comparator, outcome, context) structure for formulating research questions [16]. One checklist [17] formed part of the Support Tools for evidence-informed Policy-making (STP) [18]. While the identified checklists appeared valuable for considering applicability and transferability, none provided the detail that we sought, and there was little evidence of their use during systematic reviews.

Having been unable to identify any existing tool which met our requirements, we developed the items of the framework via scrutinising data during an examplar systematic review of integrated care models. We report the methodology underpinning development of the framework here, rather than focussing on reporting the methods and findings of the systematic review. If required, the protocol for the review providing full details of the methods employed is available via the PROSPERO database (https://www.crd.york.ac.uk/prospero), registration number: 42016037725 and the findings are reported in detail elsewhere [19]. The review examined a highly complex area of health services delivery, with interventions potentially acting at individual, team, organisational, or system-wide levels [20]. The topic of the review therefore encompassed many of the challenges for reporting on applicability and transferability, which have been identified.

**Fig. 1:**
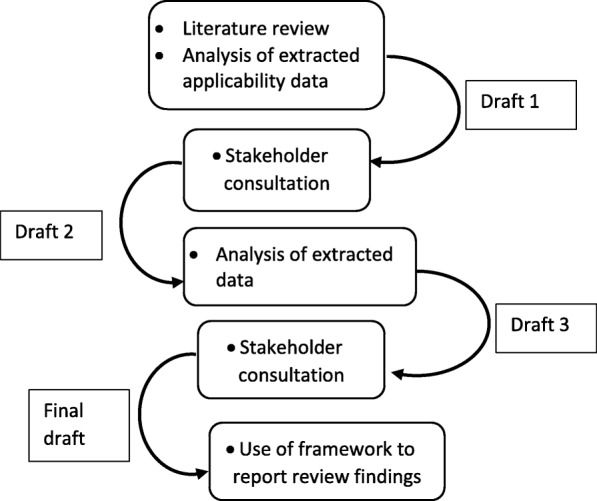
The process undertaken during development of the framework

The process of development encompassed multiple iterations of drafts and re-drafting (See Fig. 1).

Drawing on previous tools, our initial version of the framework was based on the PICOC categories. We copied and pasted text from extraction forms during our exemplar review, to a table with categories of population, intervention, comparator, outcomes, and context. We found that data which had been extracted was providing only limited detail necessary for examining applicability and transferability. We therefore went back to the studies to seek further author-reported or reviewer-identified factors which may influence applicability or transferability. In considering additional detail to extract we drew on the definitions outlined above of applicability and transferability pertaining to “insights into whether and how an intervention may be implemented or similar outcomes achieved elsewhere in a particular context”. Reference to applicability was often not part of the results reported, but was described in methods or discussion sections. Identifying this information required reviewers to specifically seek out these contextual elements during data extraction. Additional extracted text was added to our table of data within the PICOC categories.

In order to provide the detailed framework we sought, we re-examined the extracted data using principles of conventional content analysis to derive additional categories directly from the textual data [21]. The items of the draft framework were therefore based on elements drawn from existing tools, together with information reported by study authors in our exemplar review.

We sought feedback on this first draft of the framework from users of systematic reviews (five senior-level health professionals, five commissioners, six patient representatives) to identify whether our items were comprehensive and meaningful. Several changes were recommended, including the merging of organisation and systems elements, and adding additional items to the commissioning category. Other items were also suggested including “financial viability”, and “staff roles”. In response to this feedback we further refined and developed the framework to produce a second version. We returned to the extracted data (and where necessary the source documents) to examine the extent to which revised items in this second draft of the framework were reported, and to add any additional data. We then sought further stakeholder feedback on this third draft. As there were few required modifications to this version, we then used the tool as an additional method of analysis and reporting of applicability and transferability across studies during our reporting of the review findings.

## Results

The Framework for Implementation Transferability Applicability Reporting (FITAR) comprises seven main elements relating to: patients and populations; organisations and systems; financial and commissioning processes; systems leadership; features of services; features of the workforce; and elements of the interventions/initiatives. It is intended to aid detailed consideration of transferability and applicability within the overall evidence. The full framework is provided as Additional file 1.

In the following sections we describe our reflections and experiences in regard to each element of the framework during a systematic review of integrated care interventions. Brief examples relating to each element are outlined, with further examples and detail of the sources of the data provided within Additional files 1, 2, 3 and 4.

### Patients and populations

Consideration of patients and populations was a common feature of existing tools. The detailed itemisation of the framework however, highlighted the importance of ensuring that sufficient data regarding study populations were obtained during the data extraction process.

#### Patient types and conditions

We found that the framework provided a structured approach to identifying and scrutinising data which may be in narrative form or contained in study discussion sections, and that it encouraged consideration of many different sub-population characteristics. For example during our review, we identified data suggesting differential effectiveness between conditions, and the potential benefit of targeting interventions to particular populations. These data were important when interpreting the evidence within our review, as the results overall had suggested a lack of effectiveness.

#### Level of severity of conditions

While severity may have a considerable influence on the implementation and outcomes of interventions, we found considerable variance in included studies regarding the level of detail provided. Many provided little information apart from a diagnostic label, and use of the framework highlighted this as an important gap in the evidence. We found data suggesting that the costs of an intervention might be greater in patients with more severe conditions, and therefore highlighted in our reporting that severity might be important when considering the applicability of the cost effectiveness evidence.

#### Levels of deprivation

This item prompted detailed consideration of the characteristics of included study populations and socio-economic features, and we found that it alerted reviewers to data which suggested the potential for interventions to reduce or widen health inequalities. An example within our review was qualitative data, suggesting that patients who lived in difficult circumstances benefitted the most from integrated care. In our recommendations we highlighted that differential effects required consideration in future research.

#### Socio-economic diversity

This element of the framework links to levels of deprivation, but identifies a need to consider variance within a study population. We noted during extraction of population data whether studies had been carried out in areas of deprivation, or in populations which included both deprived and more affluent areas, although this item did not yield information of particular value to our synthesis.

#### Rural versus urban populations

Information regarding the geographical area was not often included by authors, but the name of the city or region was a helpful indicator of location. This item was found to be of importance during our synthesis as we noted that much of the included research had been carried in large cities (particularly London amongst the UK studies). We also identified data which reported that different models of integrated care are required in rural areas from urban areas. Our reporting of the findings therefore highlighted the need to consider whether potentially effect interventions could feasibly be transferred to settings which were not large cities.

#### Population density

While detail regarding population density was rarely reported, we found data suggesting that density of population could be important in the successful introduction of interventions. As above, this was important to include in consideration of the transferability of potentially effective interventions.

#### Level of health needs

While this item may relate to socio-economic deprivation, it could also be used to identify studies which had participants with multiple or complex conditions. In our review we did not find any suggestion of linkages between health needs, and implementation or local outcomes however, this item of the framework may be useful to highlight where evidence does not exist.

#### Prevalence of condition

We found that there was infrequent reporting of condition prevalence in primary studies however, an example of data found in one study detailed a particularly high prevalence of a target disease in their study population. While this item contributed little to the analysis or reporting during our review, it may be of value in other work.

#### Other patient characteristics

This item of the framework provided a means to collect data on any other characteristics reported in the included literature. An example of data extracted during our review was a report of age differences in usage of health services. During our reporting we were careful to include detail of the study populations when considering and comparing outcomes, and noted that much existing research had been carried out in populations of older adults. This led to our conducting a comparison of effectiveness data from studies in older adults versus other age groups.

### Features of organisations

Items relating to organisational features were largely derived from studies included in our review rather than existing tools. This may be due to the particular topic, which looked at service delivery changes, in which organisational systems and structures were influential.

#### Size of organisations

The size of organisations often had to be assumed from author report, rather than being clearly outlined, and relevant data was often descriptive rather than numerical. An example of data within this item was from a qualitative study which found that organisational features such as turnover and size of catchment area were perceived as influential in the degree of success achieved. This item of the framework influenced reporting of our review, as it highlighted that successful scaling up of potentially effective interventions cannot be assumed, and interventions in larger organisations may not be feasible in small organisational contexts.

#### Number and type of organisations

We found inconsistent reporting of other elements of the organisational environment. Our review included grey literature, and it was often reports rather than journal papers which had sufficient space to provide this potentially important detail. An example of data included here was a report of organisational culture being influenced by the type of organisations involved. As above, we highlighted in our reporting that characteristics of the organisation may be important in determining applicability and transferability.

#### Historical relationship between organisations

We found some reporting of organisational relationships, although this was scarce. An example of data noted in this item, was from qualitative studies which outlined the influence of past relationships during the development of interventions/initiatives. These data added to the emphasis in our reporting on the importance of considering the organisational environment during the introduction of interventions, as it potentially influences both implementation and outcomes.

#### Geographical proximity of organisations

This item links to the urban versus rural settings framework element. While detail of geographical location tended to be under-reported in primary studies, an example of data we noted was a study author conclusion that geographical proximity aided implementation. In our narrative synthesis we therefore distinguished where the evidence was from studies in the same hospital, the same city, or same region.

#### Baseline performance of the organisations

While we identified little data of importance within this item for our review, an example was the suggestion from one study that the standard of organisational performance from which interventions start, may have a bearing on their effectiveness. We found that the item may therefore be useful to alert reviewers to evidence from services which are rated as being particularly good or poor.

#### Policy environment at the time of introduction of the initiative

This item was suggested by our stakeholder group, who emphasised the importance of particular local or national policies. An example of data noted here was the suggestion that changing government policy had acted as an obstacle to implementation of an initiative. We found that these data highlighted a need to consider the historical timing of research studies when reporting our review findings, and during our synthesis we were careful to emphasise where evidence was from a policy context that may have limited current relevance.

#### Other changes being made con-currently

This item of the framework further recognises the need to consider the timing of research studies when synthesising findings. Examples of data which were found during our review referred in particular to funding changes. As above, during the narrative we highlighted where the financial context of studies might make applicability and transferability of interventions problematic.

#### Particular elements of infrastructure or services

This item of the framework encouraged identification of any additional contextual factors within organisations. An example of data which we noted here, was a description of the negative impact of changes to the way that patients were admitted to hospital.

### Financial and commissioning processes

Items in this category were largely suggested by our stakeholder group, who included people with budgetary responsibilities. Many elements in this category have limited applicability to studies conducted outside the UK, and the framework therefore emphasises the need to fully consider how the international literature may or may not transfer between differing healthcare financial systems.

#### Sources of funding

Funding for the initiative was often identifiable only via author funding acknowledgments. An example of data in this item were author reports of challenges in sustainability of funding. This impacted on the nature of the available evidence, as longer term follow up data had not been possible to obtain. When we were summarising the quality of the evidence, we recognised restrictions on the availability of long term evaluative data, and the challenges of ongoing funding.

#### Commissioning and budget arrangements

Detailed budgetary arrangements were rarely provided by authors. Example data within this item was a report of the impact of reconfigured commissioning and budget arrangements on the implementation of new interventions. As above, we highlighted that this often limited the quality of the evidence available.

#### Availability and ring-fencing of resources to support interventions

A further item in this category encouraged detailed consideration of the influence of available financial support on the introduction of interventions/initiatives. An example of data noted here was an author report of a particular funding stream around the time of the introduction of interventions. During the synthesis we noted where outcomes may have been achieved within a particularly positive context for their introduction.

#### Incentives

This item also highlighted the need to consider the influence of differing healthcare funding systems. An example of data in this item was an author report of beneficial effects from bonus payments in health systems in the United States. In reporting effectiveness data during our review, the importance of differing financial and commissioning contexts was recognised, and we carried out separate analyses of data from international and UK studies. The two sets of results were compared and contrasted to report where similarities and differences were apparent.

### Systems leadership

Systems leadership items were predominantly derived from the included studies, although the stakeholder group also emphasised the importance of identifying these data. The organisational nature of interventions in our review may have led to led to the detail and range of these aspects identified. These items could possibly could be collapsed into a single item in other reviews.

#### Dedicated project manager/managerial leadership roles

Detail regarding leadership of interventions varied considerably between studies, often it was little reported. Data we identified however, emphasised the quality of systems leadership as an important aspect in the success of interventions, and we highlighted this factor during the reporting of factors potentially influencing successful implementation and outcomes.

#### Managerial or clinical leadership

Information regarding leadership was frequently difficult to elucidate due to limited study reporting. Examples of data in this item were a description of managers and leaders holding a dual clinical role, and a description of external mentoring from management consultants. Use of external consultants was highlighted during the narrative findings, as this potentially influenced transferability to other settings.

#### Project champions

Champions were mentioned by several studies, although it is possible that other interventions included champions but this was not reported. Example data in this item was one author concluding that having a champion was helpful for successful implementation. In our reporting we therefore noted where use of a champion had been reported.

#### Awareness of the initiative amongst patients

We found surprisingly little reporting of patient engagement during the planning or introduction of interventions in our review. Whether this had occurred but was not outlined by the authors, or had not been carried out could not be determined. An example of data in this item was an author report that patient engagement had been a significant feature of their successful service transformation. In the reporting of our review we emphasised this as a sizeable gap in the evidence.

#### Support for the initiative amongst patients

We found that while studies typically evaluated the views/satisfaction levels amongst patients (and sometimes carers) about the quality of care they had received, there was little reporting regarding changes to service delivery. In this item we noted an example of data indicating potential conflict between patient wishes and new interventions, and also data suggesting differences between patient and carer views. In the review findings we highlighted the predominance of measurement of patient care outcomes and the dearth of evidence regarding service delivery evaluation amongst patients.

### Types of services

The type of service was an item drawn from existing tools, but further detail regarding individual items was added from our literature.

#### Location of the initiative

We found surprisingly little reporting of service location characteristics, despite this potentially having considerable influence on the transfer of findings between particular contexts. In order to explore this element of the framework in our review findings, we grouped evidence from interventions/initiatives in hospital, community, and social care settings, and investigated whether we could detect trends in outcomes between locations. Our finding of little variation in effectiveness between contexts was an important conclusion to draw.

#### Alignment with other initiatives

Only a few studies described whether other initiatives had been introduced in parallel to the integrated care intervention. While we found little data, this item alerted reviewers to where this detail was provided, and may be more useful for other reviews.

#### Standard of existing care

We found few references to standards of services in the locations being studied. An example of data noted within this item was an author reference to interventions being less effective in contexts when good practice is already being followed. While this item was of limited value for this review it may be helpful for others to consider.

### Features of the workforce

Workforce elements were identified predominantly from our included literature. As highlighted before, the organisational nature of our target interventions may have added detail to this category which may be unnecessary for other reviews.

#### Levels of motivation/support

Reviewers needed to pay close attention to this element of the framework during data extraction, as levels of motivation could be implied rather than clearly outlined. An example of data here were the many reports of challenges in gaining support from GPs and specialists. In our reporting we highlighted the key importance of gaining support for an initiative amongst staff.

#### Employment conditions

Some of the larger-scale interventions had potential to create changes in employment conditions, and we were alert to reports of these from, for example re-configured roles. An example of data noted in this item was a description of posts being funded at “more than the going rate”. We reported in our findings that employment conditions were important to consider when introducing large-scale initiatives.

#### Working location

Identifying the location of initiatives was particularly important for our review, as authors emphasised that co-location of staff is a central element of integrated care and promotes effective communication. During the review we therefore endeavoured to identify the individual elements of each integrated care intervention (such as working locations), and analyse by components and outcomes. We found that our proposed analysis proved to be too challenging however, due to limited reporting of the elements of these complex initiatives.

#### Specialist staff

We endeavoured to identify the staff that were involved in initiatives, with variable levels of success across the studies. In some reports we found examples of specialised staff being required. We noted in our review findings where there was a requirement for these staff to be available or where additional training may be required or new roles would need to be created.

#### Professions involved

We examined whether the implementation and outcomes of initiatives might be affected by the nature of different professional groups. An example of data in this item was a study which highlighted that the cost of a new intervention could differ according to the personnel profile. Overall however, we identified limited data relating to this item.

#### Size of staff group

We found that information regarding staffing levels was often difficult to ascertain. There was no indication in the literature we identified regarding optimal numbers of staff required to implement and deliver successful integration, although we noted one report that a large multi-disciplinary staff group was required.

#### Staff training

The need for staff to be trained to enable them to implement initiatives was emphasised in our included literature. An example of data in this item was report of the challenge of providing training to nursing home staff. We drew attention to the potential for interventions in nursing home to be adversely affected by this factor.

### Features of the initiatives/interventions

The initiative or intervention was a category which has been identified in the initial PICOC framework. Analysis of the included studies using the framework however, provided additional detail on the features of interventions which might be influential.

#### Complexity of initiatives

While most healthcare interventions may be described as being complex, they may vary considerably in their level of complexity. We found reference to the complexity of implementing some models of integration outweighing their potential advantages, and the potential for simple, single-faceted interventions to make more rapid progress. In our analysis we therefore grouped studies into complex (multi-element) versus simple (single element) interventions, and examined whether there was any difference in effectiveness between these types. Our finding that complex initiatives may be more successful, added additional insight to our review reporting.

#### Full versus partial integration

The majority of studies in our review evaluated some form of partial rather than full (whole organisational) integration, and we were therefore unable to draw conclusions regarding differential effectiveness, although this item may be useful during other reviews.

#### Breadth of reach

In our review we found little data relating to this item, although some studies reported that their inclusion criteria had reduced the reach of the initiative.

#### Longevity of the initiative

This item of the framework required consideration of the extent to which programmes have evolved from earlier changes. In our review we found little reporting of this information, although we noted data referring to the need for interventions to overcome initial operating problems before becoming embedded. This was important to highlight given the relatively short term nature of much of the evidence.

## Discussion

We have outlined the process of development of a framework, to support the analysis and reporting of applicability and transferability across studies during the systematic review process. We have described our experiences of using this framework during a systematic review of complex healthcare interventions, and described examples of data which were influential in our analysis and reporting. We believe that the framework is a useful addition to other available tools, and addresses needs reported in the literature [1, 5]. Our call for greater analysis and reporting of applicability and transferability echoes innovations in the field of implementation science, which has a similar focus on promoting the uptake of research findings [22].

We found that employment of the framework throughout all phases of a systematic review provided a helpful supplement to existing review methods. At the data extraction stage it helped the review team to identify an array of factors which may not be typically reported. We found that the framework alerted reviewers to data which may have been missed had we been using only a standard extraction template, and it enhanced consistency in extraction of context-related detail [23]. In our review for example we found that it prompted consideration of potentially important data regarding population characteristics, and sub-group differences, and features of interventions such as having champions and a climate of staff support. Data extracted using the framework provided additional explanatory value to our evaluation of quality of the evidence, for example highlighting the limited opportunities for longer term evaluation due to financial and commissioning changes.

At the analysis and synthesis stage, data within the framework prompted us to carry out detailed scrutiny of contextual elements across studies within our findings. For example data suggesting variance led to additional sub-group analysis in regard to age group, service location, and geographical area, which provided further insights into the evidence. Other analyses which were prompted by data within our framework explored potentially differing outcomes based on characteristics of the intervention, and characteristics of the organisation and geographical location. We argue that these supplementary analyses added to standard synthesis approaches, by providing additional information for stakeholders.

At the reporting stage the framework supported the highlighting of where information regarding applicability and transferability was present or absent in the evidence. While some items elicited little data of relevance, we would argue that the presence of these items proved a prompt to focus attention during data collection and analysis. Reporting absent features from the literature may assist interpretation of the review findings, particularly given the importance of differentiating absence of evidence, from evidence of absence [24]. Use of the framework highlighted the gap in evidence in regard to patient involvement in service transformation, it also prompted us to fully consider contextual elements such as the historical timing of studies during our reporting, and it informed reporting of similarities and differences between the national and international literature.

We suggest a number of other potential uses of the framework, which will form the basis of further work. The framework may have potential to guide the searching process during systematic reviews, by highlighting additional avenues for exploration as part of a cluster methodology [25]. A further extension of the method is to use the framework to examine the features of more, versus less successful interventions.

In addition to its use by systematic review teams, the framework has potential for use in improving the reporting of primary studies, which in turn would facilitate the future examination of factors relating to applicability and transferability within systematic reviews. We found many items of the framework where limited reporting made identification of relevant information limited. The items identified in the framework may be a useful supplement to the TIDieR (template for intervention description and replication) checklist which aims to improve the reporting of interventions [26].

Another potential use for the framework may to facilitate the involvement of stakeholder communities in a structured way during the review process. While the framework was not developed for the purpose of stakeholder engagement, we envisage that a tool such as FITAR, might have value during stakeholder involvement activities to guide discussion of the review findings.

While we found the framework a useful means to structure data extraction, suggest avenues of analysis, and provide additional detail in reporting across studies, other authors are critical of tools such as this. Atkins et al. [9] are critical on the grounds that a single universal checklist is not feasible. Other authors [1] have similarly criticised tools with set criteria as being unlikely to be useful or usable. A recent evaluation of 11 tools concluded that none were ideal to assess applicability, [27] and the authors reported that there was little evidence of their utility. Our study is one of few available which has provided a detailed report of use of such a tool during a systematic review, and provides examples of insights that might be gained.

There has been a call for a focus on mechanisms of action, rather than the production of applicability checklists [27]. Our exemplar review drew on our previous work [28] and included a logic model to visualise complex pathways of change [29]. Reflecting on this output (Additional file 4) alongside the FITAR checklist, there are several similarities, in particular in regard to influencing factors within the model. Further work to explore the combining of applicability and transferability elements within pathways of change models would be a valuable future direction.

### Limitations

One limitation of the framework may concern the lack of consideration of theory and theoretical mechanisms. While this may prove a useful addition in future versions, these considerations may be best addressed via separate systematic review methods such as realist synthesis [30]. The checklist may already be perceived by some as lengthy, counter-indicating the addition of further substantive items. We have not provided guidance regarding how data collected within each item should be analysed, leaving authors to consider how evidence should be interpreted, and the relative importance of each element within their overall study findings and conclusions.

We acknowledge that the length of the checklist adds time and resource to the data analysis process. At a time when rapid reviews are being increasingly common, [31] this may be seen as overly time consuming. If we could demonstrate that greater consideration of applicability enables review products to be more usable, this would suggest that additional time for analysis is fully justified. We have suggested that the items of our framework may have been influenced by the topic of our review, and some yielded limited information. Reviewers may find that some items within categories may be redundant when carrying out reviews of other topics.

Stakeholder involvement was a particular feature of development of the tool, although we acknowledge that this might have been improved by a wider range of stakeholders. We acknowledge the need for formal evaluations of frameworks such as the one reported here and propose this as the basis of future work. Our framework was developed at the same time as carrying out the review which provides strength in that it was grounded in data, but did not enable us to formally evaluate its usage.

## Conclusions

There is a need for an increased emphasis on applicability and transferability during systematic reviews, in order to enhance usability of research for stakeholders. We outline the development of a framework for analysing applicability and transferability which was employed during a review of complex health care service delivery interventions. We found it a useful addition to support data extraction and synthesis, and to enhance reporting of evidence, although further evaluation of tools such as this is required.

## Additional files

Additional file 1: Examples of data from our review of integrated care initiatives. Further examples for each element of the framework drawn from the studies included in the exemplar review (DOCX 16 kb)

Additional file 2: The extraction form. The form used for extracting data during the review (DOCX 53 kb)

Additional file 3: The FITAR framework. The completed framework in full (DOCX 147 kb)

Additional file 4: Logic model outlining the integrated care pathway of change. A logic model which formed one output of the exemplar review of integrated care initiatives (DOCX 41 kb)

## Abbreviations

FITAR: Framework for Implementation Transferability Applicability Reporting
GRADE: Grading quality of evidence and strength of recommendations
PARiHS: Promoting Action on Research Implementation in Health Services
PICOC: Population, intervention, comparator, outcome, context
PRISMA: Preferred Reporting Items for Systematic Reviews and Meta-Analyses
PROSPERO: International prospective register of systematic reviews
STP: Support Tools for evidence-informed health Policymaking
TIDieR: Template for intervention description and replication
