# Supplementary material for: Towards greater understanding of implementation during systematic reviews of complex healthcare interventions: the framework for implementation transferability applicability reporting (FITAR)
Source: BMC Med Res Methodol. 2019 Apr 18;19:80. doi: 10.1186/s12874-019-0723-y (PMC6472061; doi:10.1186/s12874-019-0723-y)
Supplement: Supplementary file 1 — Examples of data from our review of integrated care initiatives. Further examples for each element of the framework drawn from the studies included in the exemplar review (DOCX 16 kb) [file 12874_2019_723_MOESM1_ESM.docx]

**Additional file 3. Examples of data from our review of integrated care initiatives**

| Framework element | Examples of data |
| --- | --- |
| 1. How do the findings apply to different types of patients and populations? | While few studies had compared outcomes for different patient groups, there was the suggestion of differential effectiveness,[1] [2] and that targeting interventions to particular populations may be beneficial.[3]  Several studies highlighted differential effects, [4-7] and reported the suggestion that levels of frailty may increase intervention costs.[7]  One study [4] reported that patients who lived in difficult circumstances were perceived by staff to have benefitted the most from integrated care.  One study reported that risk profiles of patients varied according to the society and health system in which the patients lived.[5]  The range of settings included studies in urban areas [8-15] rural or semi-rural areas [16, 17] and geographically diverse areas.[18, 19]  One study emphasised the need for differing models in rural areas.[16]  One study [20] which reported that successfully integrated healthcare may only be achieved in areas of more dense population.  Two studies in our review detailed that there were particularly high levels of health needs in their study populations, [16] [21]  Two studies described a higher prevalence of disease than the national average in their local areas.[10] [13]  Age differences in usage of health services.[22, 23] |
| 2. What organisations and systems is the evidence applicable and transferable to? | Difficulty of scaling up interventions, [19]  Size of the catchment area and turnover, had an impact on service costs and configuration.[16]  Influence of the broad environment and culture on applicability,[24] and a flatter and more responsive organisational structure facilitates integration.[20]  The influence of historical relationships between organisations.[22, 23, 25]  Studies in the same hospital, [26] the same city, or county.[2]  Four of the included nursing homes were rated as having a higher level of hospital and emergency care admissions than the norm.[27]  The importance of particular local or national policies.[17, 28, 29]  Funding changes impacting on the context surrounding the introduction of initiatives.[5, 6, 30]  Changes to admission routes or criteria in parallel to the introduction of initiatives.[8, 9, 28, 31] |
| 3. What financial and commissioning processes might influence applicability and transferability? | Funding at national, regional or local area levels.[7, 12, 17]  Funding from multiple sources. [32]  Differences in outcomes between small community and larger state funded models.[33]  The impact of reconfigured commissioning and budget arrangements on the implementation of new initiatives.[4] Commissioning arrangements had added additional complexity to implementation,[5, 6]  Decommissioning of existing services had been required.[34]  Reports of an increase in healthcare spending around the time of the introduction of initiatives.[28-30]  Initiatives had been able to access particular funding streams.[15, 35]  Bonus payments to incentivise or act as a disincentive for using particular services or models.[4, 12, 15]  The influence of incentive payments was only small.[36] |
| 4. What systems leadership elements might influence applicability and transferability? | Systems leadership an important aspect of the implementation of initiatives [4, 17, 28]  Managers and leaders holding a clinical role enabler of implementation.[13, 30]  External mentoring from management consultants was available in some instances.[37]  Champions associated with successful implementation.[2, 38]  Requirement for more than one champion to be involved in multi-site interventions.[39]  Role of patient engagement [23, 40] and this could increase the validity and sense of equity during changes.[41]  Patients could be unclear about the nature of reconfiguration.[42]  Potential conflict between patient wishes and needs, and changes which increased the effectiveness of delivery. [43] |
| 5. What features of services might influence applicability and transferability? | Training and retention of staff was reported to be particularly problematic in social care settings.[39]  Initiative had taken place during a period of significant service development.[28].  Care of particularly high standard.[11, 44] Described as being in need of improvement.[45-48]  New initiatives may be less effective in contexts when good practice is already being followed. [49, 50] |
| 6. What features of the workforce might influence applicability and transferability? | Staff had developed the initiative themselves, [38, 51]  Staff had concerns.[27]  Challenges in gaining support from GPs and specialists.[4, 31, 47]  Early adopters may have more positive attitudes to change.[52].  Changes in employment conditions creating altered staff incentives,[31] changed or extended roles [4, 28, 44, 53] or rotas.[54]  Posts being funded at more than the “going rate”.[55]  Co-location of staff is a central element of integrated care.[5, 9, 28, 54]  Link between a closer staff working location, and more effective communication.[5, 6, 28]  Requirement for specialist staff to be available.[56]  New roles might be required, such as case co-ordinators.[53, 57]  Costs could differ according to the personnel profile, and that this potentially influenced the cost effectiveness of interventions.[58]  A large multi-disciplinary team was required to deliver a reconfigured service.[59]  The need for staff to be trained to enable them to implement initiatives[27, 44]  Challenge of providing training to nursing home staff. [39] |
| 7. What elements of the initiatives might influence applicability and transferability? | Complexity of implementing some models of integration outweighing their potential advantages.[60]  The implementation of simple, single-faceted interventions make more rapid progress.[37]  Some potential for variance in effects in regard to use of services, [61] and potential cost savings.[34]  A third of potential patients were not accepted into the model.[48]  Initiatives are likely to need time to overcome any initial operating problems.[4] |
