# Supplementary material for: Towards greater understanding of implementation during systematic reviews of complex healthcare interventions: the framework for implementation transferability applicability reporting (FITAR)
Source: BMC Med Res Methodol. 2019 Apr 18;19:80. doi: 10.1186/s12874-019-0723-y (PMC6472061; doi:10.1186/s12874-019-0723-y)
Supplement: Supplementary file 2 — The extraction form. The form used for extracting data during the review (DOCX 53 kb) [file 12874_2019_723_MOESM2_ESM.docx]

**Additional file 1. The extraction form**

| **[First author + year)**  **Country:**   \| **RCT** \|  \| \| --- \| --- \| \| **Non-RCT** \|  \| \| **CBA** \|  \| \| **BA** \|  \| \| **Comparator:** \| \| \| **Length of follow up:** \| \| \| **Qualitative** \|  \| \| **Cross-sectional** \|  \| \| **Other (specify)** \|  \|   **Sample size:**  **Population characteristics:**   \| **Type of group** \|  \| \| --- \| --- \| \| **Condition/**  **department** \|  \| \| **Sex** \|  \| \| **Age** \|  \| \| **Other (specify)** \|  \|   **Context:** | **Data collection method:**  **Outcome measures:**   \|  \| \| --- \| \|  \| \|  \| \|  \|   **The intervention:** | **Summary of results:**  **Main author conclusions:**  **Reported associations or causative links:**  **Potential applicability considerations:** |
| --- | --- | --- | --- | --- | --- | --- | --- | --- | --- | --- | --- | --- | --- | --- | --- | --- | --- | --- | --- | --- | --- | --- | --- | --- | --- | --- | --- | --- | --- | --- | --- | --- | --- | --- |
