# Supplementary material for: Towards greater understanding of implementation during systematic reviews of complex healthcare interventions: the framework for implementation transferability applicability reporting (FITAR)
Source: BMC Med Res Methodol. 2019 Apr 18;19:80. doi: 10.1186/s12874-019-0723-y (PMC6472061; doi:10.1186/s12874-019-0723-y)
Supplement: Supplementary file 3 — The FITAR framework. The completed framework in full (DOCX 147 kb) [file 12874_2019_723_MOESM3_ESM.docx]

**
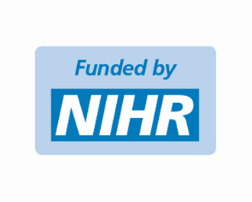

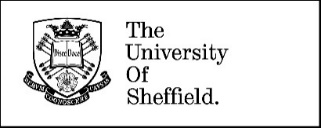
Additional file 2. The FITAR framework**

**FRAMEWORK FOR IMPLEMENTATION TRANSFERABILITY APPLICABILITY REPORTING (FITAR)**

**Seven questions to consider potential applicability and transferability of the overall evidence**

**1. How do the findings apply to different type of patients and populations?**

**2. What organisations and systems is the evidence applicable and transferable to?**

**3. What financial and commissioning processes might influence applicability and transferability?**

**4. What systems leadership elements might influence applicability and transferability?**

**5. What features of services/s might influence applicability and transferability?**

**6. What features of the workforce might influence applicability and transferability?**

**7. What elements of the initiatives might influence applicability and transferability?**

| **1. HOW DO THE FINDINGS APPLY TO DIFFERENT TYPES OF PATIENTS AND POPULATIONS?** |
| --- |
| **What type of conditions were included?** |
| **What was the level of severity of these conditions?** |
| **Was the level of deprivation in the study populations particularly higher/lower than the national average?** |
| **Was the level of socio-economic diversity in the study populations particularly high/low?** |
| **Was the research carried out in particularly rural or urban areas?** |
| **Was the research carried out in particularly compact or geographically spread regions with high or low populations/density?** |
| **Were the levels of health needs in the research populations higher/lower than the national average?** |
| **Was the prevalence of a condition particularly low or high in the populations researched?** |
| **2. WHAT ORGANISATIONS AND SYSTEMS IS THE EVIDENCE APPLICABLE AND TRANSFERABLE TO?** |
| **What was the size of the organisations in the studies (e.g. population served, size of catchment area, number of patients, turnover, range of services)?** |
| **Was the initiative within one, or across different organisations?** |
| **What type of organisations were involved e.g. health/social care/private/voluntary?** |
| **Was there a historical relationship/existing alignment between the services and/or organisations?** |
| **What was the geographical proximity of services (such as multiple hospitals)?** |
| **Was the baseline performance of the study organisations higher/lower compared to the national average?** |
| **What was the policy environment at the time of introduction of the initiative, were there particular drivers/levers for this change?** |
| **Were other changes being made con-currently?** |
| **Was there audit/an evaluation loop in place?** |
| **Were there particular elements of infrastructure in place within the organisations studied, such as existing shared IT systems?** |
| **Did the organisations studied have other relevant services in place (e.g. having an intermediate care team)** |
| **Did the organisations studied have particular admission routes (e.g. Medical/surgical, GP-referred, via emergency and urgent care)** |
| **3. WHAT FINANCIAL AND COMMISSIONING PROCESSES MIGHT INFLUENCE APPLICABILITY AND TRANSFERABILITY?** |
| **What was the source of funding for the initiative, was it ring fenced?** |
| **What were the commissioning/budget arrangements in the organisations studied?** |
| **Were available resources reduced/increased around the time of introduction?** |
| **Were there incentives for organisations to be included?** |
| **Any other key elements of transactability or financial viability?** |
| **4. WHAT SYSTEMS LEADERSHIP MIGHT INFLUENCE APPLICABILITY AND TRANSFERABILITY?** |
| **Was there a dedicated project manager/managerial leadership role, was leadership from managerial or clinical staff?** |
| **Was there a project champion?** |
| **Was there engagement and support for the initiative amongst patients?** |
| **5. WHAT FEATURES OF SERVICES MIGHT INFLUENCE APPLICABILITY AND TRANSFERABILITY?** |
| **What was the location for the initiative (specialist versus non-specialist unit, outpatients versus inpatients, acute versus community, vertical or horizontal integration)?** |
| **Had other initiatives already been introduced in the setting researched, was there alignment between initiatives?** |
| **Was the care or service in the locations studied, of a particularly poor or good standard?** |
| **6. WHAT FEATURES OF THE WORKFORCE MIGHT INFLUENCE APPLICABILITY AND TRANSFERABILITY?** |
| **What level of motivation/support for the initiative was there amongst the workforce in the research?** |
| **What was the level of willingness to change/take part amongst the workforce in the research?** |
| **What were the employment conditions for the staff involved in the research (e.g. same/different employers, type of contract, employed for the project or transferred)?** |
| **What was the working location of staff involved in the research (e.g. co-location in same office, same site, travelling between sites)?** |
| **Was there a requirement for specialist staff (e.g. GP with special interest)?** |
| **Which professions/staff roles were involved in the initiatives reported?** |
| **What size of staff group was involved (for example small or large teams)?** |
| **What training was required/provided?** |
| **7. WHAT ELEMENTS OF THE INITIATIVES MIGHT INFLUENCE APPLICABILITY AND TRANSFERABILITY?** |
| **What were the components of the interventions reported - was it simple or with multiple elements?** |
| **Was the integration full or partial?** |
| **Was the intended reach narrow or broad (e.g. inclusion criteria, number and type accepted/not accepted into initiative)?** |
| **How long had the initiative been in place?** |
| **Are there specific requirements that must be in place?** |
