# Supplementary material for: Towards greater understanding of implementation during systematic reviews of complex healthcare interventions: the framework for implementation transferability applicability reporting (FITAR)
Source: BMC Med Res Methodol. 2019 Apr 18;19:80. doi: 10.1186/s12874-019-0723-y (PMC6472061; doi:10.1186/s12874-019-0723-y)
Supplement: Supplementary file 4 — Logic model outlining the integrated care pathway of change. A logic model which formed one output of the exemplar review of integrated care initiatives (DOCX 41 kb) [file 12874_2019_723_MOESM4_ESM.docx]

**Additional file 4. Logic model outlining the integrated care pathway of change**

**Workforce focus**

MDTs

MDT meetings

Professional role change

Relocation staff

Co-location staff

Working patterns

Single employer

Single line management

Leaders/champions

**Organisation & system focus**

Integrated patient records

Shared IT system

Single entry point

New units

Care groupings

Services in community

PROCESS CHANGE

INFLUENCING FACTORS

OUTCOMES

**Resource usage**

Number of clinician contacts

GP appointments

Length of stay

Unscheduled admissions

Admissions/no. inpatients

Elective admissions

Re-admission

A&E attendance

Out-patient appointments

Prescribing rates

Access to resources

**Quality of care**

Perceived quality

Quality standards

Patient satisfaction

Patient preferences met

Time in A&E

Number incidents/complaints

Length of wait (contact, diagnosis, investigation, treatment)

Perceptions of quality

Access to services

Unmet need identified

**Staff work experience**

Community care activity

Secondary care activity

Healthcare utilisation

Cost of provision

SYSTEM-WIDE IMPACTS

**Patient role**

Relationship with health care provider

Patient understanding of treatment

**Care provision**

Discharge planning

Timeliness/flow/co-ordination of care

Continuity of care

Variance in practice/adherence to guidelines

Duplication

Practitioner time

**Information**

Shared information

Accuracy/completeness of recording of information

Shared knowledge

**Organisational structure and processes**

Reconfigurations

Governance and accountability

Financial arrangements

Holistic/patient centred care model

Common values/beliefs/

priorities amongst staff/

organisations

Relationships between staff

Relationships between organisations

Trust/support between staff

Staff mutual understanding

Communication between staff

TARGET AREAS FOR CHANGE

**Patient-care focus**

Patient education

Agreed referral criteria

Joint assessment

Common assessment

Pathways/protocols

Care co-ordinator

Integrated assessment and treatment

Joint patient review/discharge

INTERVENTION ELEMENTS

**Financial and governance focus**

Joint commissioning

Financial integration

Team budget

Organisational integration

**Patient-related**

Focus of change

Patient engagement

Sharing of patient information

**Workforce-related**

Emotional response to change

Professional identity

Professional role boundaries

Stability of workforce

Employer

Power and hierarchies

History of co-operation

Provision of training

Staff involvement/engagement

Employer

**Management and leadership**

Local leaders/champions

Effective leadership

Support for innovation

**Organisation & system-related**

Capacity/resources

IT systems/logistics

Clarity of vision

Organisational culture

External threats/policy context

Audit/evaluation

Commissioning

Budgetary/financial

Governance
